# Optimizing Adjuvant Stereotactic Radiotherapy of Motor-Eloquent Brain Metastases: Sparing the nTMS-Defined Motor Cortex and the Hippocampus

**DOI:** 10.3389/fonc.2021.628007

**Published:** 2021-02-26

**Authors:** Yvonne Dzierma, Michaela Schuermann, Patrick Melchior, Frank Nuesken, Joachim Oertel, Christian Rübe, Philipp Hendrix

**Affiliations:** ^1^ Department of Radiotherapy and Radiation Oncology, Saarland University Medical Centre, Homburg, Germany; ^2^ Department of Neurosurgery, Saarland University Medical Centre and Saarland University Faculty of Medicine, Homburg, Germany

**Keywords:** nTMS mapping, motor cortex, hippocampus sparing, treatment planning, functional optimization, IMRT, stereotactic radiation, brain metastases

## Abstract

Brain metastases can effectively be treated with surgical resection and adjuvant stereotactic radiotherapy (SRT). Navigated transcranial magnetic stimulation (nTMS) has been used to non-invasively map the motor cortex prior to surgery of motor eloquent brain lesions. To date, few studies have reported the integration of such motor maps into radiotherapy planning. The hippocampus has been identified as an additional critical structure of radiation-induced deficits. The aim of this study is to assess the feasibility of selective dose reduction to both the nTMS-based motor cortex and the hippocampi in SRT of motor-eloquent brain metastases. Patients with motor-eloquent brain metastases undergoing surgical resection and adjuvant SRT between 07/2014 and 12/2018 were retrospectively analyzed. The radiotherapy treatment plans were retrieved from the treatment planning system (“original” plan). For each case, two intensity-modulated treatment plans were created: the “motor” plan aimed to reduce the dose to the motor cortex, the “motor & hipp” plan additionally reduce the dose to the hippocampus. The optimized plans were compared with the “original” plan regarding plan quality, planning target volume (PTV) coverage, and sparing of organs at risk (OAR). 69 plans were analyzed, all of which were clinically acceptable with no significant differences for PTV coverage. All OAR were protected according to standard protocols. Sparing of the nTMS motor map was feasible: mean dose 9.66 ± 5.97 Gy (original) to 6.32 ± 3.60 Gy (motor) and 6.49 ± 3.78 Gy (motor & hipp), p<0.001. In the “motor & hipp” plan, dose to the ipsilateral hippocampi could be significantly reduced (max 1.78 ± 1.44 Gy vs 2.49 ± 1.87 Gy in “original”, p = 0.003; mean 1.01 ± 0.92 Gy vs. 1.32 ± 1.07 Gy in “original”, p = 0.007). The study confirms the results from previous studies that inclusion of nTMS motor information into radiotherapy treatment planning is possible with a relatively straightforward workflow and can achieve reduced doses to the nTMS-defined motor area without compromising PTV coverage. Furthermore, we demonstrate the feasibility of selective dose reduction to the hippocampus at the same time. The clinical significance of these optimized plans yet remains to be determined. However, with no apparent disadvantages these optimized plans call for further and broader exploration.

## Introduction

Brain metastases can effectively be treated with surgical resection and/or stereotactic radiotherapy (SRT). Lesions located within or adjacent to critical motor areas pose a challenge to both the neuro- and radiosurgeon. Increasing survival from effective interdisciplinary treatment regimens shifts attention to ameliorated secondary outcome rates such as improved motor function, cognitive function and quality of life. Preoperative neurosurgical planning and surgical resection itself predominantly aim to identify and preserve critical motor areas. In the past decade, navigated transcranial magnetic stimulation (nTMS) has been used to non-invasively map the motor cortex prior to surgery of motor eloquent brain lesions. Here, these preoperative motor maps appear to facilitate better resection rates while maintaining neurological function (1–5).

Whereas planning of SRT focusses on sparing distinct structures at risks, motor-eloquent areas have not routinely been integrated. Motor deficits have been observed to occur after high-dose Gamma Knife SRS to sites close to the motor cortex (6). Beyond direct motor-deficits, Pfeiffer et al. (7) have postulated a relationship between higher dose to the precentral gyrus and impaired verbal and working memory, attention and executive functions. To date, a small number of studies have reported the integration of motor maps into radiotherapy planning for dose reduction to motor areas, primarily in CyberKnife and GammaKnife treatment (8–11). Two studies (12, 13) have been carried out for linear accelerator (linac)-based radiotherapy, one of which considered patients with brain metastases. All studies showed that the inclusion of nTMS information into the radiotherapeutic planning workflow was possible and allowed for improved dose sparing of the motor-eloquent areas. However, additional confirmation from different medical centers and using different planning systems is still warranted. Furthermore, the hippocampus has been identified as an additional critical structure of radiation-induced cognitive deficits, which has not been considered in previous studies on nTMS in radiotherapy. In addition to the observed negative impact of whole-brain radiotherapy on cognitive outcome (14, 15), several studies have focussed on the hippocampus itself as one of the most critical structures for radiation injury owing to the ongoing neurogenesis in the subgranular zone of its dentate gyrus (16–25). Higher dose to the hippocampus has particularly been associated with impaired verbal memory and higher executive functions (20, 25). Thus, hippocampal sparing in brain radiotherapy planning has also recently gained significant attention.

The aim of this study is hence to confirm the implementation of nTMS motor information into treatment planning of linac-based stereotactic radiotherapy of motor-eloquent brain metastases, and to assess the feasibility of selective dose reduction to both the nTMS-based motor cortex and the hippocampi at the same time.

## Materials and Methods

Patients with motor-eloquent brain metastases undergoing nTMS-based surgical resection and adjuvant SRT between 07/2014 and 12/2018 were retrospectively analyzed. Metastases were regarded as motor-eloquent when infiltration of the precentral gyrus and/or pyramidal tract was presumed, or if the precentral gyrus and adjacent sulci could not be distinguished due to neuroanatomical distortion.

### nTMS Mapping and Import Into the Radiotherapy Treatment Planning System

Patients received pre-operative magnetic resonance imaging (MRI) on a 1.5 T or 3 T scanner (Magnetom Symphony-TIM 1.5 T, Magnetom Skyra 3.0 T, Siemens, Erlangen, Germany) with contrast-enhanced T1-weighted MPRage in the axial direction (repetition time TR = 0.9 ms, echo time TE = 3.52 ms, flip-angle 15°, slice thickness 1 mm), on which dataset navigated transcranial magnetic stimulation was performed. The nTMS motor mapping was carried out using the Nexstim NBS system 4.3 (Nexstim Oy, Helsinki, Finlany) as previously described (5, 26, 27). In brief, the patients were sitting reclining in a chair with open eyes with surface electromyography electrodes attached to the muscles used for mapping (m. first dorsal interosseus, abductor pollicis brevis, abductor digiti minimi). The presumed location of the hand knob was used as a starting point, then varying the coil location and orientation to determine the resting motor threshold (RMT), defined as the lowest stimulus intensity which will elicit a 50 μV peak-to-peak amplitude motor evoked potential in five out of ten stimulations. The hand area was then mapped using 110% of the RMT and 0.25 Hz, holding the coil perpendicular to the precentral gyrus. Where possible, the lower extremity was mapped as delineated by the anterior tibial and plantar muscles, using 130% RMT intensity and a coil orientation perpendicular to the midline/falx. However, since lower extremity mapping was not available for all patients in this study collective, we only included patients who suffered from a motor-eloquent lesion where imaging predominantly appeared to demonstrate jeopardy of the upper extremity/hand area. Consequently, retrospective dose planning was carried out only for sparing of the upper extremity motor area.

In all cases the nTMS motor maps for the upper limb (i.e. hand area) were exported as an additional secondary dataset into the original radiotherapy treatment plan in the Philips Pinnacle treatment planning system (TPS) V16.2 for each patient. The secondary image was rigidly co-registered to the primary data set (planning computed tomography (CT)) and planning MRI (acquired post-operatively in both T1 MPRage and T2 flair weighting) based on a mutual information algorithm and then manually shifted until optimal correspondence was achieved. Correspondence was verified independently by two radiation physicists and/or radiation oncologists. The original clinical target volume (CTV), planning target volume (PTV) and organs at risk (OAR: lenses, bulbi, optic nerves, chiasm, cochleae, brainstem) for brain irradiation as defined on the planning-CT and planning-MRI were re-checked and the additional organs at risk (OAR’s) were contoured (i.e. nTMS-based motor cortex, basal ganglia, thalamus, hippocampus) based on the T1 weighted planning MRI sequences according to (28, 29). Each hippocampus was expanded by 5 mm into all directions to create the “hippocampus avoidance zone” for plan optimization.

### Treatment Planning

For the original plans, different radiotherapy techniques were used: static beams with 3D-conformal radiotherapy (3D-CRT), intensity-modulated radiotherapy (IMRT) or non-coplanar arcs (30); 6 MV or flattening-filter-free 7 MV photons were used (31, 32).

For each metastasis, two re-optimized treatment plans were created in addition to the original clinically treated plan (“original”). The “motor” plan spared the hand motor areas delineated by nTMS. The “motor & hipp” plan aimed to reduce the dose to the nTMS-based motor cortex and the hippocampi. If the plan was originally planned by IMRT or by 3D-CRT, the same beam arrangements were used for the re-optimization; if the original plan used conformal non-coplanar arcs, a new plan was established by IMRT planning with between 8 and 13 beams. The maximum number of segments allowed was 35. The optimization objective for the motor cortex was to lower the maximum as well as the mean dose as much as possible without reducing the coverage of the PTV. Regarding the hippocampi, the optimization objectives were iteratively reduced to lower the maximum and the mean dose as much as possible without reducing the coverage of the PTV or burden the motor cortex again.

Optimization was performed using direct machine parameter optimization (DMPO) on a 0.2 cm dose grid; the final dose distributions were calculated using a collapsed cone (CC) convolution algorithm. All plans were revised by an experienced radiation oncologist and were in agreement with the general guidelines of the DEGRO Working Group on SRS for clinical stereotactic treatment (33) and the dose limits for sensitive brain structures based on the criteria of the Quantitative Analysis of Normal Tissue Effects in the Clinic (QUANTEC, 34).

### Plan Evaluation

To evaluate plan differences, several measures of quality are considered.

The Paddick conformity index (CI) (34–36)

$$CI=OR\cdot UR=\frac{TV_{PIV}^{2}}{PIV\cdot TV}$$

is the product of the Paddick overdose ratio (OR) and underdose ratio (UR).

The overdose ratio

$$OR=\frac{TV_{PIV}}{PIV}$$

estimates the ratio of the PTV volume inside the prescribed 80 % isodose (TV_PIV_) to the total volume encompassing the 80% isodose (PIV = V_80 %_). This relates the covered PTV volume to the total volume irradiated with the prescribed encompassing dose.

The underdose ratio

$$UR=\frac{TV_{PIV}}{TV}$$

estimates the ratio of the PTV volume inside the 80 % isodose (TV_PIV_) to the PTV volume (TV). This relates the covered PTV volume to the total PTV volume.

The homogeneity index (HI) (34–36)

$$HI=\frac{PTV_{1\%}-PTV_{99\%}}{PTV_{mean}}$$

measures the PTV homogeneity by considering *PTV*_1%_ as a measure of the maximum and *PTV*_99%_ as a measure of the minimum dose in the PTV.

The gradient index (GI)

$$GI=\frac{V_{40\%}}{PIV}$$

indicates the steepness of dose fall-off by comparing the volume of the prescription encompassing isodose to the volume (80%) of half this dose (V_40%_).

Two important values are V_12 Gy_ and V_10 Gy_ as well as their relative value to the total brain volume, as they correlate with the risk for necrosis in the case of stereotactic radiosurgery (37).

Besides these plan quality parameters, the doses in the critical sensitive brain structures based on the QUANTEC recommendations (38) as well as the PTV are determined. For the PTV, D_01 %_ is given as a measure of the relative maximum; it is considered relative to the prescription dose in target point to estimate the amount of overdosage. D_99%_ of the PTV is considered as a measure of the relative mimimum PTV dose, it is considered relative to the prescribed encompassing dose of 80% to estimate the amount of underdosage.

For the motor cortex, the intersections with the PTV, 90 %, 80 %, 70 %, 50 % and 20 % isodose are determined. Furthermore, D_01%_ as a measure for the maximum dose and the mean dose are evaluated for the motor cortex and hippocampus as well as the other OARs.

### Statistical Analyses

Dose-volume histogram (DVH) values were exported by an in-house Pinnacle script. Each OAR as well as the motor cortex and the PTV were saved in a CSV (comma separated variables) table. The reorganization into one table for each OAR and all calculations were performed with MATLAB R2019b. A normal distribution could not be presumed, so Wilcoxon’s signed-rank test for paired data was used. A 5 % level of significance was applied. For multiple comparisons (three scenarios), a Bonferroni correction was applied, in which p values below 0.0167 were considered statistically significant.

## Results

A total of 52 patients were identified. Of these, 24 patients received stereotactic radiation therapy at this department. The remaining patients either received a different radiotherapy regimen or received radiotherapy elsewhere. For all but one patient, the 80% isodose of the prescribed maximum dose in the isocentre was required to encompass the PTV. One patient received a different fractionation with the 95% isodose level surrounding the target volume and was therefore excluded from the analysis. Another patient received irradiation for three neighboring target volumes which were jointly optimized – this patient was also excluded, since re-optimization would have involved all three target volumes with different prescriptions and isocenters. Treatment details for the remaining 22 patients included in the analysis are given in **Table 1**.

**Table 1:** Patient characteristics.

Number of patients/cases	22/23
Age (average, range) [years]	65.1 (45–86)
Sex (female/male)	11/11 (50.0/50.0%)
Location (pre-/postcentral)	15/8 (65.2%/34.7%)
Paresis preoperatively	15 (68.2%)
Ø BMRC rank preoperatively	4.2 ± 0.7 (3–5)
Paresis post-operative day 7 Resolved Improved (but not resolved) Unchanged Deteriorate (new or worse)	12 (54.5%) 3 (20.0% of 15) 8 (53.3% of 15) 12 (54.5% of 22) 0 (0%)
BMRC rank post-operative day 7	4.4 ± 0.6 (3–5)
Paresis post-operative day 60 Resolved Improved (but not resolved) Unchanged Deteriorate (new or worse)	4 (18.2%) 11 (73.3% of 15) 2 (13.3% of 15) 10 (45.5% of 22) 0 (0%)
BMRC rank post-operative day 60	4.8 (4–5)
CTV/GTV volume (mean, range) [cm³]	9.5 ± 10.3 (0.6–36.5)
PTV volume (mean, range) [cm³]	16.4 ± 16.0 (2.1–56.7)
Prescription to the isocenter	
1 x 2,500 cGy 1 x 2,250 cGy 1 x 1,800 cGy 3 x 1,125 cGy 5 x 625 cGy	2 (8.7%) 3 (13.0%) 1 (4.3%) 6 (26.1%) 11 (47.8%)
Radiotherapy technique	
IMRT (7–21 beams) Conformal arcs (7–9 arcs) static beams 3D-CRT (7-20)	11 (47.8%) 6 (26.1%) 6 (26.1%)
Motor cortex inside PTV If yes, percent of motor cortex outside PTV If no, minimum distance (mm)	9 (39.1%) 90.1 ± 6.6 (76.9–98.6) 8.3 ± 8.5 (0–31.5)

CTV, clinical target volume; GTV, gross tumor volume; GTV were used in case of single-fraction treatment (stereotactic radiosurgery, SRS), whereas CTV were used for fractionated stereotactic radiation therapy SRT; in both cases, expansion was performed to create the PTV, planning target volume; IMRT, intensity-modulated radiation therapy; 3D-CRT, 3 dimensional conformal radiotherapy; BMRC, British Medical Research Council.

**Figure 1:**
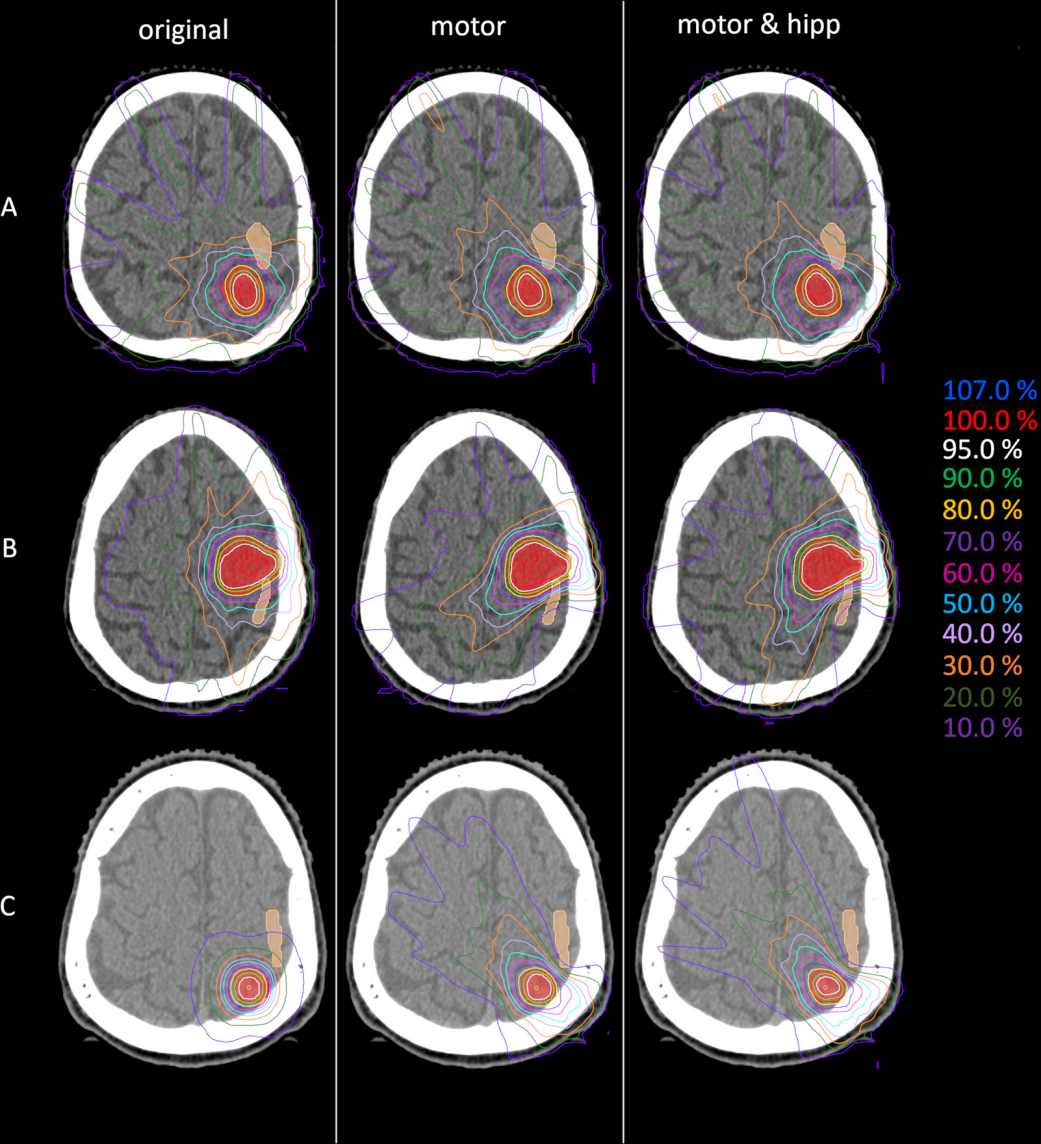
Example dose distributions for the original (left), motor (middle) and motor&hipp (right) plans for three different patients and planning scenarios. **(A)** patient treated originally by static beams, **(B)** patient treated originally with intensity-modulated radiotherapy (IMRT), **(C)** patient treated originally with non-coplanar arcs. The planning target volume (PTV) is delineated by the red filled contour, the nTMS-based motor cortex in skin color.

For all 22 patients, the “motor” and “motor & hipp” treatment plans were considered acceptable for treatment. An example of the resulting isodose distributions is shown in **Figure 1**. Metrics for plan quality and dose to organs at risk are given in **Table 2**.

**Table 2:** Plan evaluation using metrics for plan quality, planning target volume (PTV) coverage, and dose to organs at risk.

	Original	Motor	Motor & hipp	p 1-2	p 1-3	p 2-3
CI	0.767 ± 0.106	0.789 ± 0.087	0.784 ± 0.089	0.287	0.523	0.503
	(0.524–0.907)	(0.609–0.932)	(0.586–0.908)			
OR	0.801 ± 0.115	0.797 ± 0.095	0.792 ± 0.095	0.761	0.484	0.523
	(0.524–0.932)	(0.610–0.946)	(0.587–0.938)			
UR	0.959 ± 0.047	0.991 ± 0.021	0.991 ± 0.020	**0.001**	**0.001**	0.689
	(0.843–1.000)	(0.901–1.000)	(0.903–1.000)			
HI	1.276 ± 1.894	1.512 ± 1.517	1.439 ± 1.450	0.465	0.465	0.055
	(0.198–8.381)	(0.171–4.306)	(0.178–4.024)			
GI	3.775 ± 0.897	4.129 ± 1.046	4.357 ± 1.122	0.024	**0.003**	**0.003**
	(2.555–6.700)	(2.990–7.314)	(3.044–7.252)			
PTV						
D_01%,rel [%]_	102.43 ± 5.84	102.57 ± 1.65	102.59 ± 1.55	**0.014**	0.030	0.858
	(99.81–128.64)	(99.76–104.70)	(99.76–104.80)			
D_99%,rel [%]_	97.61 ± 5.92	103.20 ± 4.57	102.72 ± 4.53	**0.001**	**0.001**	0.273
	(83.72–109.72)	(86.16–108.60)	(85.48–108.64)			
nTMS motor map						
D_01%_ [Gy]	18.762 ± 9.734	16.895 ± 10.198	16.895 ± 10.148	**0.002**	**0.002**	0.661
	(1.82–33.24)	(1.68–34.23)	(1.11–33.33)			
D_mean_ [Gy]	9.659 ± 5.972	6.319 ± 3.596	6.493 ± 3.784	**<0.001**	**<0.001**	0.162
	(0.906–19.559)	(0.348–15.008)	(0.294–15.800)			
Hippocampus ipsilateral						
D_01%_ [Gy]	2.493 ± 1.870	3.426 ± 2.468	1.775 ± 1.440	**0.003**	**0.003**	**<0.001**
	(0.08–7.36)	(0.09–9.67)	(0.09–4.99)			
D_mean_ [Gy]	1.320 ± 1.074	1.818 ± 1.718	1.005 ± 0.918	**0.002**	**0.006**	**<0.001**
	(0.039–3.458)	(0.051–6.595)	(0.051–2.747)			
Hippocampus contralateral						
D_01%_ [Gy]	0.723 ± 1.106	0.736 ± 1.148	0.562 ± 0.801	0.402	0.554	0.302
	(0.05–4.84)	(0.04–4.63)	(0.04–3.04)			
D_mean_ [Gy]	0.323 ± 0.396	0.238 ± 0.314	0.204 ± 0.207	0.648	0.648	0.951
	(0.021–1.439)	(0.022–1.462)	(0.023–0.744)			
Hippocampus avoidance zone ipsilateral						
D_01%_ [Gy]	3.027 ± 2.775	4.316 ± 3.186	2.433 ± 2.191	**<0.001**	0.019	**<0.001**
	(0.10–13.15)	(0.11–14.21)	(0.11–10.06)			
D_mean_ [Gy]	1.338 ± 1.078	1.794 ± 1.597	1.044 ± 0.944	**0.002**	**0.006**	**<0.001**
	(0.042–3.665)	(0.053–5.700)	(0.053–3.183)			
Brain without PTV						
D_12Gy_ [cm³]	41.37 ± 34.88	40.15 ± 29.21	44.28 ± 34.02	0.378	**0.011**	**<0.001**
	(3.72–110.91)	(6.52–111.55)	(7.05–120.41)			
D_10Gy_ [cm³]	57.04 ± 47.08	56.04 ± 39.32	61.95 ± 46.57	0.394	**0.008**	**<0.001**
	(6.06–157.31)	(9.71–141.69)	(10.76–177.80)			
D_01%_ [Gy]	15.920 ± 7.148	16.472 ± 5.867	16.806 ± 5.928	0.191	0.026	**0.004**
	(1.11–25.31)	(3.46–24.24)	(3.60– 24.35)			
D_mean_ [Gy]	2.000 ± 1.176	2.140 ± 1.093	2.173 ± 1.112	0.024	**0.004**	0.447
	(0.543–4.130)	(0.749–4.395)	(0.750–4.327)			
Brainstem						
D_01%_ [Gy]	1.489 ± 1.300	2.088 ± 1.843	1.254 ± 1.120	0.029	0.191	**0.001**
	(0.06–5.20)	(0.08–5.99)	(0.06–4.67)			
D_mean_ [Gy]	0.483 ± 0.480	0.543 ± 0.644	0.366 ± 0.350	0.078	0.378	**<0.001**
	(0.029–1.782)	(0.035–2.604)	(0.030–1.442)			
Thalamus ipsilateral						
D_01%_ [Gy]	3.482 ± 3.271	4.472 ± 3.565	3.234 ± 2.825	**0.010**	0.280	**0.001**
	(0.14–14.07)	(0.16–11.43)	(0.12–10.02)			
D_mean_ [Gy]	1.730 ± 1.628	2.185 ± 2.130	1.610 ± 1.593	0.033	0.033	**0.001**
	(0.079–6.643)	(0.086–7.771)	(0.077–6.296)			
Basal ganglia ipsilateral						
D_01%_ [Gy]	6.073 ± 7.368	6.813 ± 7.374	6.733 ± 7.481	0.128	0.161	0.733
	(0.17–26.79)	(0.17–27.00)	(0.15–27.01)			
D_mean_ [Gy]	1.963 ± 2.131	2.413 ± 2.795	2.315 ± 2.683	0.144	0.201	0.236
	(0.064–8.024)	(0.077–9.065)	(0.066–8.587)			

Average ± standard deviation (min-max). After Bonferroni correction, p values below 0.017 were considered statistically significant and are marked in bold. Lenses, cochleae, bulbi, optic nerves, chiasma, contralateral hippocampus avoidance zone, contralateral thalamus and basal ganglia are not listed. For these organs, the dose was far below the clinical acceptable limits, most comparisons did not show statistically significant differences and no clear tendencies in improved sparing could be observed. CI, conformity index; OR, overdose ratio; UR, underdose ratio; HI, homogeneity index; GI, gradient index.

### PTV and Organs at Risk in the Re-Optimized Plans

**Figure 2:**
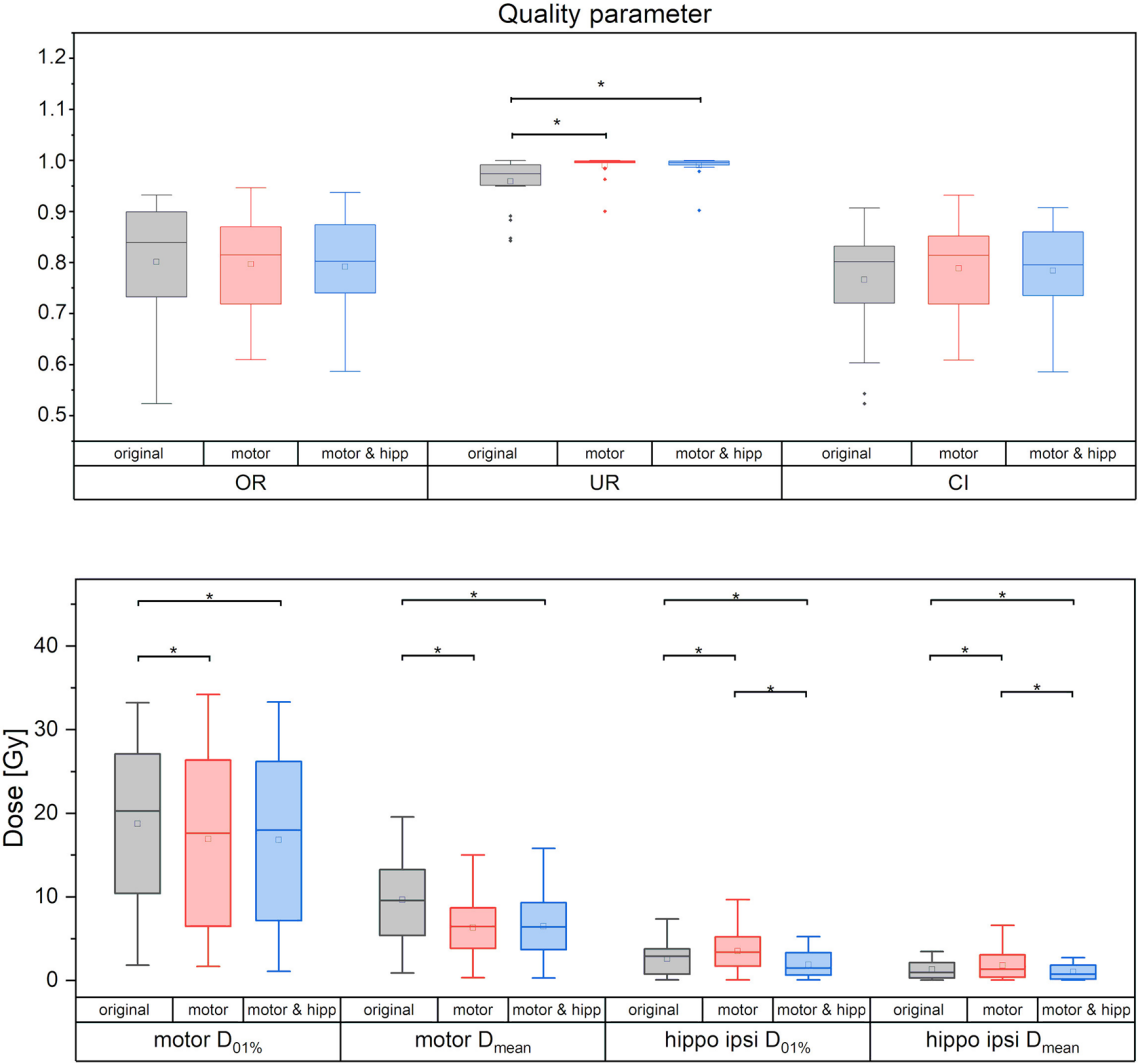
Quality measures of planning target volume (PTV) coverage (upper panel) and dose to motor cortex and ipsilateral hippocampus. Statistically significant differences are denoted by asterisk.

No significant differences between the “original”, “motor” and “motor & hipp” plans were observed for the coverage of the planning target volume as assessed by the conformity index, PTV minimum and maximum, and overdose ratio (**Figure 2**). There was a small, but statistically significant improvement in the underdose ratios of the “motor” and “motor & hipp” plans when compared with the original plans (but not with each other). The gradient index assessing dose fall-off outside the PTV was slightly worse in the newly-optimized plans, however, this did not affect the clinical acceptability of the plans.

All organs at risk could be well protected in both the “motor” and “motor & hipp” plans. The volume of the brain receiving a dose of 10 Gy or 12 Gy was slightly increased in the “motor & hipp” plans relative to the original and “motor” plans by ca. 3–4 cm³. However, this parameter is only relevant for stereotactic radiosurgery, i.e. very high single dose fractionation regimes. If we consider only patients receiving stereotactic radiosurgery (five cases), the three planning scenarios do not exhibit a significant difference in the volume of the 12 Gy isodose inside healthy brain, although there appears to be a trend toward somewhat increased volume (9.2 ± 2.8 cm³ and 9.5 ± 2.4 cm³ in the “motor” and “motor & hipp” plans, respectively, compared with 5.9 ± 1.9 cm³ in the original plan, p = 0.0625 and p = 0.3125, respectively). Simultaneously, sparing of the hippocampus resulted in reduced dose to the brainstem, thalami and basal ganglia. Some variations in OAR sparing were observed between the planning scenarios, however, all these organs received very little dose as compared with the planning objectives remained far below the clinically acceptable limits.

### Sparing of the Motor Cortex

**Figure 3:**
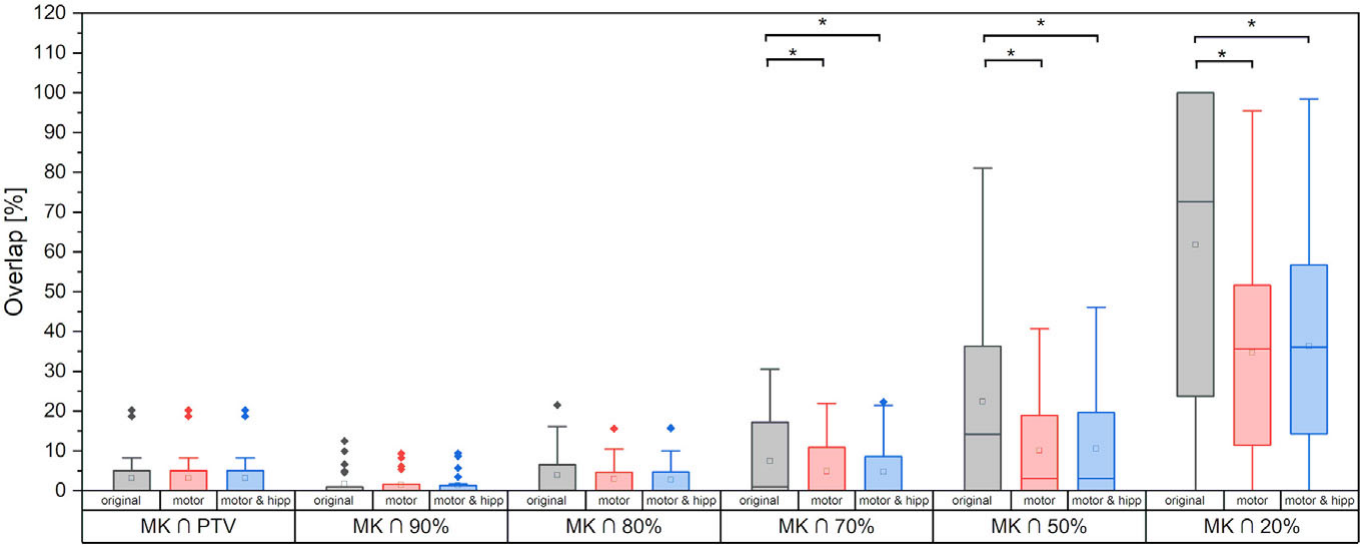
Motor cortex volume included inside planning target volume (PTV) and different relative isodose lines. Statistically significant differences are denoted by asterisk.

Sparing of the motor cortex could significantly be improved by both scenarios with nTMS information included, with mean dose to the motor cortex reduced from 9.66 ± 5.97 Gy (original) to 6.32 ± 3.60 Gy (motor) and 6.49 ± 3.78 Gy (motor & hipp), respectively (p<0.001 for both re-optimized plans vs. “original”). Regarding the spatial relationship of the motor cortex with the isodose levels (**Figure 3**), a reduction of overlap with all isodoses from 20 to 90% of the prescribed dose was observed relative to the original plans; however, this difference only reached statistical significance for the 70% isodose, becoming more pronounced for the lower isodose levels. The volume of the nTMS-derived motor map covered inside the 70% isodose could hence be reduced from an average of 7.4% (max 30.5%) to 4.8% (max 21.9%) (p = 0.015). A larger reduction was observed for the volume contained within the 50% isodose (22.3% vs. 10.1%, p = 0.003) and 20% isodose (61.8% vs. 34.7%, p < 0.001).

**Figure 4:**
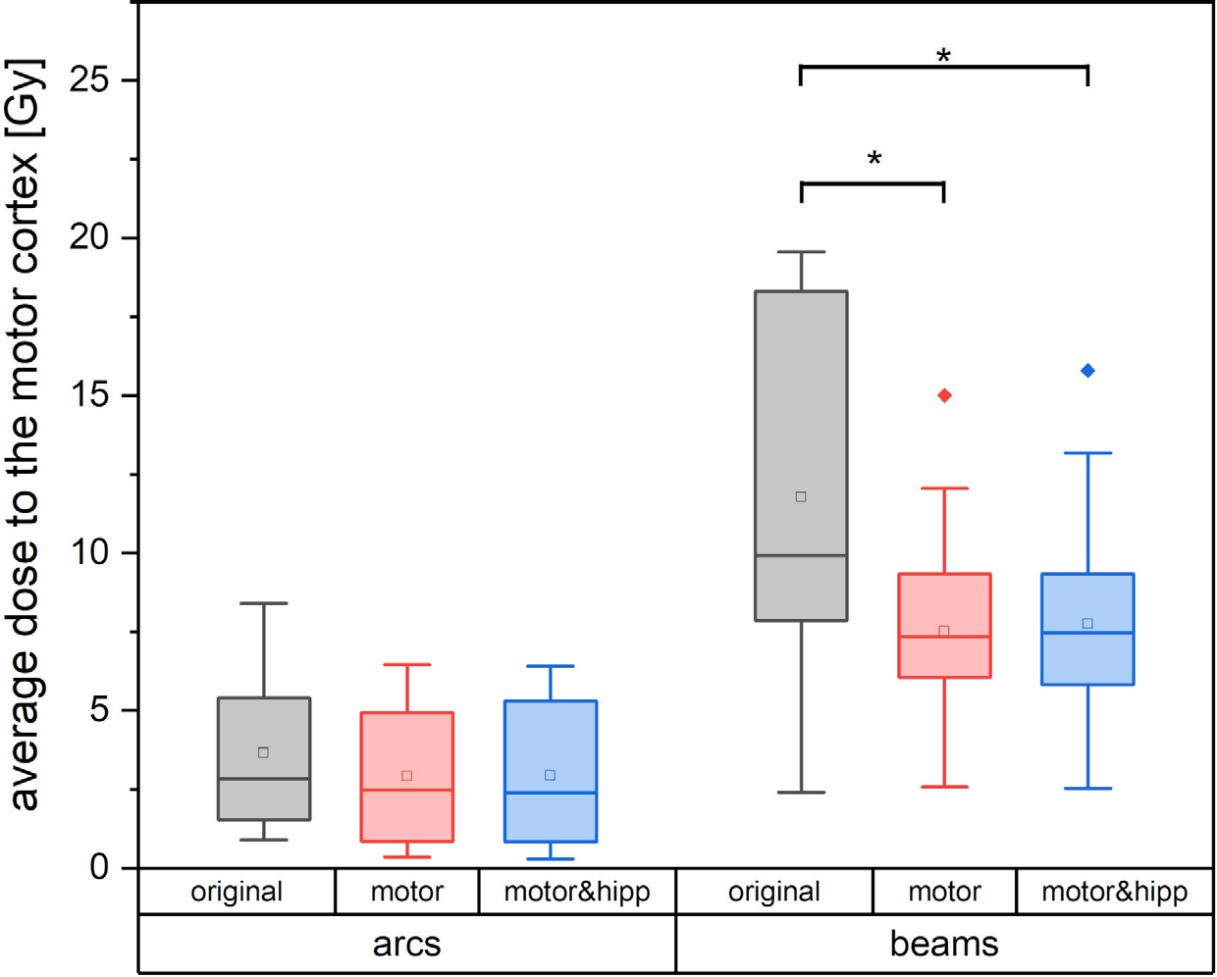
Average dose to the motor cortex for plans using non-coplanar conformal arcs vs. plans with static beams or intensity-modulated radiotherapy (IMRT). Statistically significant differences are denoted by asterisk.

Since the patients in the collective received the original radiation treatment using three different technical approaches (3D-CRT, IMRT, non-coplanar arcs), a separate analysis of the dose to the motor cortex is performed for planning techniques relying on static beams with the original geometry (“beams” plans including 3D-CRT and IMRT) and non-coplanar “arcs” plans. Dose to the motor cortex in the “motor” and “motor & hipp” plans is compared with the original plans separately for the “beams” and “arcs” techniques in **Figure 4**. A considerably greater improvement could be attained by re-optimizing the “beams” plans than the “arcs” plans. This can also been visually confirmed in the isodose distribution (**Figure 1**). In all cases, the re-optimized plans show a more asymmetric behavior than the original plans since the isodoses are deformed so as to avoid the nTMS volume. While the prescription isodoses still encompass the PTV at least as well as in the original plan, the intermediate isodoses show a relatively strong asymmetry, where the gradient toward the motor cortex is much steeper than in all other directions. In particular, the original “arcs” plans already had a very steep gradient from the combination of many non-coplanar arcs, which cannot be achieved by a relatively simple co-planar IMRT beam configuration. Therefore, this planning scenario suffers from a relatively strong change in gradient (also reflected in GI), while achieving relatively little additional sparing of the motor cortex.

For the “beams” plans, a strong positive correlation (Pearson correlation coefficient r = 0.903, p < 0.001) is observed between the distance PTV to nTMS-derived motor map and the relative change in nTMS mean dose, i.e., a greater improvement in nTMS mean dose is obtained the closer the motor cortex is located to the PTV.

**Figure 5:**
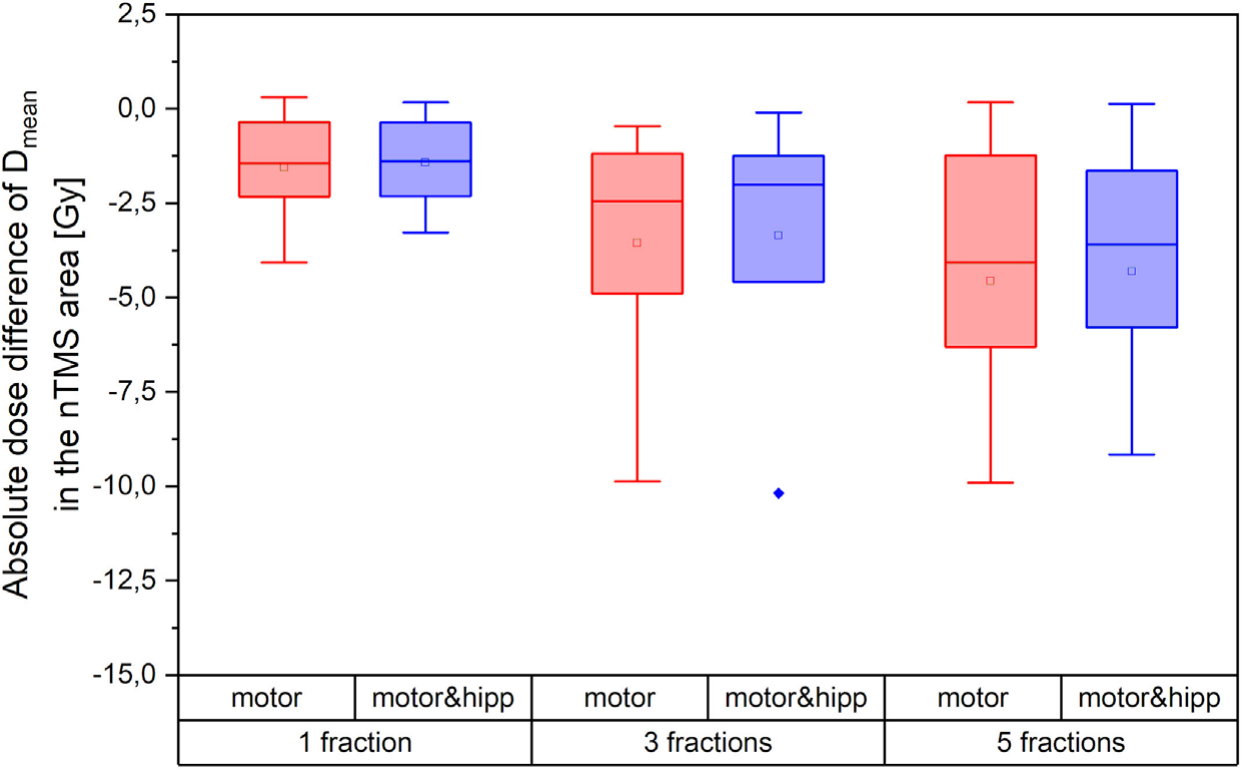
Absolute dose sparing in the navigated transcranial magnetic stimulation (nTMS) area (mean dose) achieved by the reoptimized plans as a function of fractionation regime.

Both the absolute dose reduction to the nTMS and its clinical relevance depend on the fractionation scheme. **Figure 5** therefore shows the absolute difference in nTMS mean dose achieved for different fractionation regimes (one, three, or five fractions as detailed in **Table 1**). The amount of sparing achievable was higher in absolute dose for the more fractionated schedules. The single-fraction regimes included in our collective comprised three “arcs” plans. We have already seen that the “arcs” plans provided least improvement in nTMS dose by reoptimization, which will also influence the relatively low sparing achieved by the single-fraction regimes. Still, an average dose reduction (nTMS mean dose) of 0.88 Gy (both for “motor” and “motor & hipp”) plans could be achieved for this sub-collective, and for two patients around 2 Gy decrease were reached.

### Sparing of the Hippocampus

In the “motor” plans without hippocampus sparing, the dose to the ipsilateral hippocampus was increased over the original plan (in which also no attempt at hippocampus sparing was made): maximum dose 3.43 ± 2.47 Gy vs. 2.49 ± 1.87 Gy, p = 0.003; mean dose 1.82 ± 1.72 Gy vs. 1.32 ± 1.07 Gy, p = 0.002. After reoptimization in the “motor & hipp” plans, hippocampus ipsilateral mean and maximum dose (**Figure 2**) were significantly lower, even when compared with the original plan (maximum dose 1.78 ± 1.44 Gy in “motor & hipp” vs. 2.49 ± 1.87 Gy in “original”, p = 0.003; mean dose 1.01 ± 0.92 Gy in “motor & hipp” vs. 1.32 ± 1.07 Gy in “original”, p = 0.006). Also, hippocampus contralateral mean and maximum dose could be reduced by re-optimization, though not reaching statistical significance (max dose 0.56 ± 0.80 Gy in “motor & hipp” vs. 0.72 ± 1.11 Gy in “original”; mean dose 0.20 ± 0.21 Gy in “motor & hipp” vs. 0.32 ± 0.40 Gy in “original”).

## Discussion

The study demonstrates the feasibility of selective dose reduction to both the nTMS-identified motor area and hippocampus for a comparison of treatment 69 plans, including three different planning approaches (static 3D-CRT, IMRT, non-coplanar conformal arcs). Even though this introduces some heterogeneity in the study collective, this wide selection of planning methods is representative of routine radiotherapy practice and differences could actually be identified in the amount of sparing achieved depending on the original planning technique.

The presented study cohort adds to hitherto only two reports investigating the implementation of nTMS into linac-based radiotherapy planning (12, 13), whereas the remaining studies investigated nTMS maps for CyberKnife (8, 9) or GammaKnife (10, 11) stereotactic radiosurgery. Linac-based radiotherapy, CyberKnife and GamaKnife differ in technical implementation, planning and dosimetry. As CyberKnife and GammaKnife availability is restricted to a small selection of specialized centers, whereas linear accelerators with stereotactic capability are much more wide-spread, an evaluation of motor cortex and hippocampus sparing achievable with linac stereotactic radiotherapy will be useful for a wider range of patients and treatment centers.

Diehl et al. (12) presented a planning study for 30 patients with high-grade gliomas treated by volumetric modulated arc therapy (VMAT) in a simultaneously integrated boost (SiB) concept. By including dose constraints to the nTMS motor maps (only areas outside the planning target volume), they could achieve a dose reduction of 12.8% (4.6 Gy), without compromising PTV coverage. In a collective more similar to the herein presented cohort, Schwendner et al. (13) presented a re-optimization of VMAT plans for a collective of 30 patients with supratentorial brain metastases. Again, the nTMS motor map not included in the PTV was spared in the re-optimized plans, resulting in a dose reduction of ca. 4.1 Gy (18.1%). Our study presents a similar collective of patients with brain metastases, but with a wider variation in original planning techniques, and with a larger range of distances of the motor cortex from the PTV. In line with those previous reports motor cortex sparing by about 3 Gy in mean dose (ca. 30%), with best results obtained for the static 3D-CRT and IMRT treatment plans was observed. Additionally, a correlation of dose reduction with proximity to the motor cortex was recognized.

A considerably greater improvement could be attained by re-optimizing the static 3D-CRT and IMRT plans than the plans using non-conformal arcs. However, this does not imply that non-conformal arcs should generally be the preferred planning scenario, since this is only applicable to relatively spherical and small target volumes. The dose to the motor cortex in the “beams” plans might be higher than in the “arcs” plans not primarily because of a presumably inferior planning technique, but possibly because a more complex PTV shape or volume, which might have been associated with a higher motor cortex dose and precluded the use of arcs. Yet, in the “arcs” plans, a small improvement could still be achieved by re-planning.

For the first time, the hippocampus is included as an additional organ at risk in the attempt to spare the motor map. Reducing radiation dose to the hippocampus and nTMS based motor map was feasible simultaneously without compromising the PTV or other organs at risk. Despite the inherent low dose to the ipsilateral hippocampi in the original plans (D_01%_ 2.49 ± 1.87 Gy, mean dose 1.32 ± 1.07 Gy), a further dose reduction by 23%–28% could be achieved (D_01%_ 1.78 ± 1.44 Gy, mean dose 1.01 ± 0.92 Gy). In particular, in the plans re-optimized for motorcortex sparing without hippocampus inclusion, the dose to the ipsilateral hippocampus was increased, so that the optimization of hippocampus dose reduced the dose from the “motor” plans by over 40%. Singular optimization of the motor cortex resulted in increased doses to the ipsilateral hippocampus avoidance zone and thalamus. A similar trend (without reaching statistical significance) could be found for the brainstem and ipsilateral basal ganglia. Although these changes would not have resulted in clinical rejection of the plans – partly because these structures are not routinely contoured and dosimetrically evaluated in clinical plans and partly because no clinically evaluated dose limits are yet available for these structures – the inclusion of hippocampus protection at the optimization stage could totally reverse this effect without loss of motor cortex sparing.

The external validity of the study is limited since nTMS mappings were not uniformly performed for the entire primary cortex. In a subset of included patients, lesion-specific mapping was performed to outline peri-lesional and critical motor areas to facilitate surgery. To increase internal validity, we included only nTMS-motor areas of the upper extremity into the optimization assessment. The omission of these structures means that for some patients at least the anatomic leg or face areas of the motor cortex may have received higher doses, since functional leg or face areas were not optimized in the plans. For future studies, comprehensive nTMS mapping of bilateral primary motor cortices including nTMS-based tractography is required to allow evaluation to which extent specific motor areas can be spared and whether sparing causes increased doses to other critical areas. Few authors have hitherto presented treatment plans with dose optimization for white-matter tracts (8, 9), and the clinical significance of this improvement is yet unclear.

### Clinical Significance of the Dosimetric Improvements

The clinical significance of these dose-optimized plans for motor function, cognitive function and quality of life yet remains to be elucidated. The risk of brain radionecrosis in single-session radiosurgery is correlated with the volume receiving doses of 10 Gy and 12 Gy, and motor deficits are among the complications observed (39). However, a dose threshold has not yet been definitely established for functional impairment of the motor cortex. For SRS of the corticospinal tract, Maruyama et al. (40) proposed a 5% risk of motor complication when the volume receiving 20 and 25 Gy exceeded 58 mm³ and 21 mm², respectively. Based on a neuroanatomical target theory for a patient collective treated by whole-brain radiotherapy with boost in conventional fractionation (1.8–2Gy/fraction), Pfeiffer et al. (41) proposed that cognitive outcomes were affected by the volume of the left hippocampus receiving 10 Gy and by the volume of the left precentral gyrus receiving 40 Gy, among a range of other regions.

For the hippocampus, recent radiotherapy optimization studies have aimed at reducing maximum dose to 16–17 Gy and minimum dose to 9 Gy in hippocampus-sparing whole-brain radiotherapy (14, 25). However, while a significant correlation was observed between mean dose to the left hippocampus and cognitive deterioration (25) as well as hippocampal volume reduction (41), no dose cut-off was defined in these studies. Hippocampus volume loss was observed to be significant one year after high-dose radiotherapy (> 40 Gy), but not after low-dose radiotherapy (< 10 Gy) by Seibert et al. (42). The normal-tissue complication probability (NTCP) model proposed for the hippocampus by Gondi et al. (17) suggests that an EQD_2_ dose (dose equivalent to 2 Gy fractions) of greater than 7.3 Gy to 40% of the bilateral hippocampi may be associated with memory impairment as assessed by list-learning delayed verbal recall; however, this model could not be confirmed for low-grade glioma patients in the EORTC 22033 clinical trial (21). In the Hopkins Verbal Learning Test-Revised Delayed Recall, Ma et al. (19) reported 20% probability of decline for D_100%_ hippocampus doses above 10.9 Gy and 50% probability for 59.3 Gy. Taken together, a relatively steep gradient appears to exist in this dose range, making any attempts of hippocampus sparing potentially relevant for cognitive performance.

In the present patient collective, doses to the hippocampus were already low in the original plans due to the location of the brain metastases near motor-eloquent cortical areas. However, even low doses to the hippocampus may influence neurogenesis and differentiation of the dendritic arbor, as has been shown in animal studies for single-fractions of 1 Gy (16) and fractionated low dose irradiation [5 to 20 fractions of 0.1 Gy (43)]. Although a reduction in hippocampus dose as attained in our study has not yet been proven to result in an observable change in clinical outcome, with no apparent disadvantages these optimized plans call for further and broader exploration. Prospective studies are required to assess whether dose sparing to the motor cortex and hippocampus can contribute to improved motor and/or cognitive outcomes and higher health-related quality of life.

In contrast to radiotherapy, surgical treatment of motor-eloquent lesions does not take brain areas into account that are deemed uncritical for the procedure or even contralateral. For radiotherapy, however, this information is essential due to the larger expansion of intermediate- and low-dose areas. From the neurosurgical perspective, uncritical areas may not be mapped or tractography not be performed unless explicitly requested for. The quality of the localization would be improved if post-operative mapping was performed at the same time with the planning MRI, so that a more precise fusion of the images unbiased by changes in tumor or edema distribution would become possible. On this basis, a clinical evaluation of the motor and cognitive performance could be correlated with the dose to the motor cortex and corticospinal tract, which will be a prerequisite for exploring dose-effect relationships. Furthermore, the distance of these structures from the high-dose volume may serve for risk stratification of radiation-induced effects. Rosenstock et al. (44). and Sollmann et al. (45). proposed nTMS-based risk-stratification models for neurosurgery. They correlated the proximity of motor-eloquent brain lesions to the motor cortex and corticospinal tract with the risk of motor deterioration due to treatment. Furthermore, the RMT was also reported as a marker of increased hazard for post-operative deficit. Possibly, a similar observation can be established for radiotherapy.

While exploring these possibilities, we would like to emphasize the importance of including in the considerations a critical structure such as the hippocampus, which might even be less well protected when a new optimization objective such as motor cortex sparing is added to the planning process without adequate hippocampus constraints. On-going neurogenesis in this structure renders it particularly sensitive to radiation-induced damage, so care should be taken to reduce hippocampus dose if this is possible. As we could show, sparing of the motor cortex and hippocampus is not mutually exclusive. Rather, adequate coverage of the PTV can be reconciled with combined dose reduction to the nTMS-defined hand area and hippocampus at no detriment in plan quality.

## Conclusions

Selective dose reduction to the motor cortex and hippocampus is feasible without compromising PTV coverage or other organs at risk. The inclusion of the nTMS-based information on the motor cortex into plan optimization allowed for about 30% dose reduction (approximately 3 Gy in mean dose). However, singular optimization of the motor cortex causes an increase in dose to the hippocampus, thalamus and brain stem, which can be prevented by including hippocampus dose as an additional planning objective. In these plans, a further dose reduction to the hippocampus along with the motor cortex can be achieved, resulting in increased overall protection of these functional cortical areas.

## Data Availability Statement

The raw data supporting the conclusions of this article will be made available by the authors, without undue reservation.

## Author Contributions

YD and PH designed the study concept. PH and JO recruited the patient. JO was responsible for the surgical treatment of the patients and CR for the radiotherapy. nTMS mapping was performed by PH. Patient datasets were retrieved from the data base and retrospectively revised by YD. Import into the planning system and rigid registration with the planning CT was performed by YD and MS. PM and YD contoured the organs at risk. MS created the re-optimized radiotherapy treatment plans and performed the plan evaluation and statistical analysis. PM and FN evaluated the treatment plans. YD wrote the manuscript. MS prepared the figures and tables. All authors were involved in the interpretation of the results. All authors contributed to the article and approved the submitted version.

## Conflict of Interest

The authors declare that the research was conducted in the absence of any commercial or financial relationships that could be construed as a potential conflict of interest.
